# Bortezomib-Induced Complete Heart Block and Myocardial Scar: The Potential Role of Cardiac Biomarkers in Monitoring Cardiotoxicity

**DOI:** 10.1155/2016/3456287

**Published:** 2016-01-28

**Authors:** Sachin Diwadkar, Aarti A. Patel, Michael G. Fradley

**Affiliations:** ^1^Division of Cardiovascular Medicine, University of South Florida, 2 Tampa General Circle, Tampa, FL 33606, USA; ^2^H. Lee Moffitt Cancer Center & Research Institute, 12902 Magnolia Drive, Tampa, FL 33612, USA

## Abstract

Bortezomib is a proteasome inhibitor used to treat multiple myeloma and mantle cell lymphoma. Traditionally, bortezomib was thought to have little cardiovascular toxicity; however, there is increasing evidence that bortezomib can lead to cardiac complications including left ventricular dysfunction and atrioventricular block. We present the case of a 66-year-old man with multiple myeloma and persistent asymptomatic elevations of cardiac biomarkers who developed complete heart block and evidence of myocardial scar after his eighth cycle of bortezomib, requiring permanent pacemaker placement. In addition to discussing the cardiovascular complications of bortezomib therapy, we propose a potential role for biomarkers in the prediction and monitoring of bortezomib cardiotoxicity.

## 1. Introduction

With newer chemotherapeutic agents, survival rates for many cancers have increased dramatically. Unfortunately, many of these agents have unintended cardiovascular side effects. There has been increasing focus on developing strategies with cardiac biomarkers to better predict and monitor chemotherapy-induced cardiotoxicity [1]. Although the majority of the work has focused on anthracyclines and HER2 targeted therapies, other chemotherapeutics can lead to cardiac dysfunction. Bortezomib is a proteasome inhibitor used to treat multiple myeloma and mantle cell lymphoma. Though rare, cardiac complications including heart block and heart failure have been described [2]. We report the case of a patient with persistently elevated cardiac biomarkers who developed complete heart block and myocardial scar after his eighth cycle of bortezomib therapy. This case illustrates potential bortezomib-induced cardiac complications and the possible role of cardiac biomarkers in identifying individuals at higher risk for developing bortezomib cardiotoxicity.

## 2. Case Report

**Figure 1:**
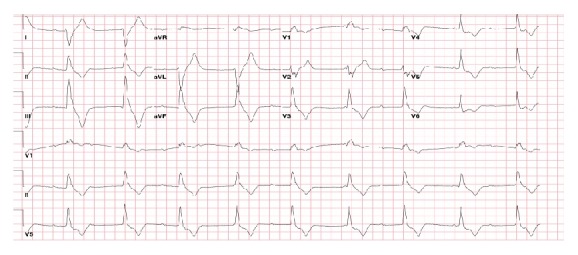
Electrocardiogram demonstrates complete heart block with a wide ventricular escape rhythm at 55 beats per minute.

A 66-year-old man with stage IIIA IgA lambda chain multiple myeloma receiving chemotherapy with bortezomib, lenalidomide, and dexamethasone (VRD) with no significant cardiovascular history presented for a routine pre-stem cell transplant clinic appointment. He was feeling well and had not experienced any recent chest pain, dyspnea, palpitations, syncope, or presyncope. He reported mild fatigue but continued to engage in daily 30-minute walks without limitation. In the oncology clinic, he underwent routine electrocardiogram as part of the stem cell transplant workup which revealed complete atrioventricular (AV) block with a ventricular escape rhythm of 55 beats per minute (bpm) (Figure 1). On exam, his blood pressure was 124/59 mmHg and pulse was 52 bpm. Physical exam revealed a regular rhythm with normal heart sounds and no other abnormalities. Laboratory testing showed normal electrolytes, normal kidney and liver function, and mild normocytic anemia (hemoglobin 13.0 g/dL). Cardiac biomarkers were elevated with troponin I of 2.49 ng/mL, CK-MB of 20.2, and creatine phosphokinase of 549. BNP was also elevated at 333 pg/mL. Echocardiogram revealed normal left ventricular systolic function with an ejection fraction of 55% and mild hypokinesis of the midinferolateral myocardium. Throughout this time, the patient remained asymptomatic. The patient was transferred urgently to a tertiary care facility for further management.

The patient was first diagnosed with multiple myeloma nine months earlier after seeking medical attention for right arm weakness and paresthesia. A bone scan was consistent with extensive metastatic disease and he underwent biopsy of a right humerus lytic lesion which demonstrated a lambda restricted plasma cell population consistent with a plasmacytoma. On serologic testing, he was noted to have an IgA lambda monoclonal gammopathy and a 24-hour urine protein was notable for 19.8 g of protein of which 19.6 g represented urine free lambda excretion. Despite his significant urinary protein excretion, his kidney function (BUN, creatinine, and glomerular filtration rate) remained normal. Bone marrow biopsy confirmed multiple myeloma without evidence of amyloid deposition. He was initiated on bortezomib, cyclophosphamide, and dexamethasone (VCD). He received three cycles without significant response, so his treatment regimen was changed to VRD. Complete treatment regimen with associated clinical and laboratory parameters is presented in Table 1.

During his initial chemotherapy regimen with bortezomib, cyclophosphamide, and dexamethasone, he was noted to have elevated troponin I at 0.163 ng/mL and BNP at 1810 pg/mL. Given these findings, he was referred to cardiology clinic for evaluation of possible cardiac amyloidosis (despite negative bone marrow evaluation). Aside from hyperlipidemia, he had no personal or family history of cardiovascular disease including significant conduction system disease. In the month prior to his multiple myeloma diagnosis, he completed an exercise stress echocardiogram which was negative for ischemia. The echocardiogram revealed normal left ventricular (LV) function and wall thickness and the stress ECG showed sinus rhythm with right bundle branch block. Due to the elevated cardiac biomarkers, he underwent a repeat transthoracic echocardiogram which again demonstrated normal LV function without significant diastolic dysfunction. A repeat ECG obtained at the time showed normal sinus rhythm with normal voltage and incomplete right bundle branch block. As a result of these findings, cardiac amyloidosis was considered unlikely.

**Figure 2:**
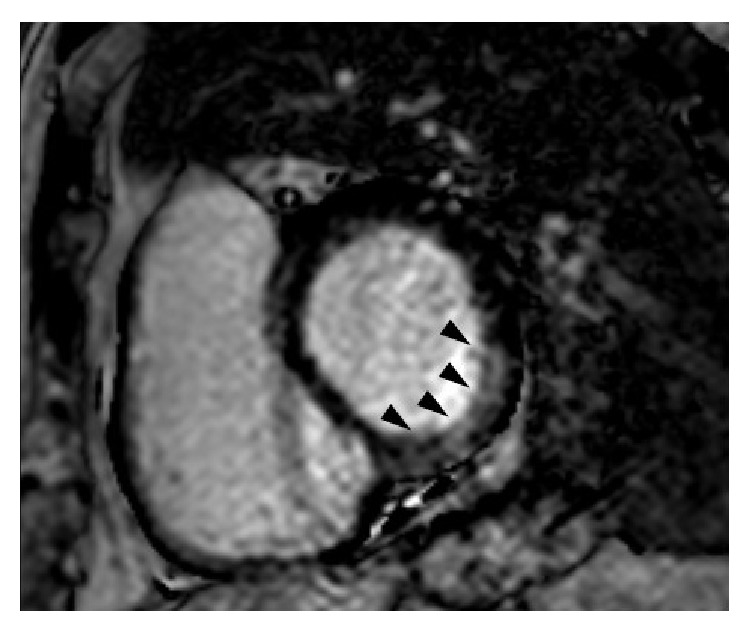
Cardiac magnetic resonance demonstrates late gadolinium enhancement (arrows) in the inferolateral left ventricle.

**Figure 3:**
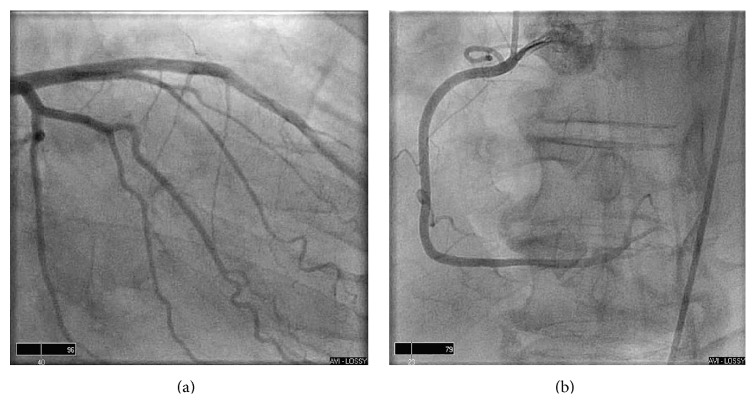
Coronary angiograms show no obstructive lesions in (a) the left anterior descending and the left circumflex artery or (b) the right coronary artery.

Upon transfer to the tertiary care hospital, the patient remained in complete AV block. A cardiac MRI was obtained, revealing normal left ventricular function (ejection fraction of 60%), with myocardial thinning and a small subendocardial perfusion defect in the midinferolateral segment with focal late gadolinium enhancement (LGE) suggestive of scar/fibrosis (Figure 2). There was no evidence on the MRI to suggest cardiac amyloidosis or other infiltrative diseases such as LV hypertrophy and/or diffuse LGE. Left heart catheterization was subsequently performed showing angiographically normal coronary arteries (Figures 3(a) and 3(b)). Throughout all of these procedures, the patient remained hemodynamically stable in complete heart block. He ultimately underwent implantation of a permanent dual-chamber pacemaker. Unfortunately, he was unable to move forward with stem cell transplant after the pacemaker implantation due to significant pulmonary fibrosis identified during vital organ testing. He elected not to receive any additional chemotherapy and eventually expired from progression of his multiple myeloma.

## 3. Discussion

This case demonstrates rare adverse cardiovascular effects of bortezomib and introduces the potential usefulness of biomarkers to predict cardiac dysfunction in patients undergoing active treatment with this chemotherapy. Utilizing cardiac biomarkers such as troponin I and BNP as a method to predict cardiac dysfunction during treatment with bortezomib has not been previously described.

Our patient presented with atrioventricular (AV) heart block in the setting of ongoing chemotherapy with bortezomib (8 cycles). Advanced age and cumulative dose have been shown to increase the risk of adverse cardiac events [3]. In our case, the patient was noted to have asymptomatic elevations of troponin and brain natriuretic peptide (BNP) as early as six months prior to the development of AV block on ECG. These levels were persistently elevated, with troponin I reaching levels suggestive of myocardial necrosis. Further evaluation with cardiac MRI demonstrated scar/fibrosis in the inferolateral left ventricle, and there was no significant coronary artery disease seen on left heart catheterization. Based on a Naranjo score of 5-6, it is “possible or probable” the scar seen on MRI and the AV block was an adverse event due to bortezomib exposure [4].

Chemotherapy-induced cardiotoxicity is the likely cause for the observed findings. The patient underwent thorough cardiac evaluation to rule out common cardiac conditions that can cause the observed findings such as atherosclerotic heart disease and myocarditis. In addition, based on the echocardiogram and cardiac MRI findings, there is no evidence to suggest cardiac amyloid or other infiltrative diseases as the cause of his cardiac dysfunction. Cardiac MRI has emerged as a noninvasive method to aid in diagnosis of cardiac amyloidosis in preselected patients with studies showing sensitivity and specificity in the range of 86–88% and 86–90% in those with positive endomyocardial biopsy [5, 6]. In patients without biopsy proven amyloidosis (endomyocardial or other tissue types), those with cardiac amyloidosis demonstrate characteristic diffuse LGE patterns coupled with other markers of cardiac dysfunction such as LV hypertrophy [7]. Our patient had a negative bone marrow biopsy for amyloidosis and cardiac MRI lacked typical features of amyloidosis, suggesting extremely low likelihood of cardiac amyloidosis.

Although the patient was exposed to multiple chemotherapeutics, we believe that bortezomib is the likely cause of his cardiac dysfunction. Cyclophosphamide can lead to left ventricular dysfunction, myopericarditis, and heart block; however, these findings generally occur during therapy or the first several weeks posttreatment [8, 9]. When our patient's cardiac dysfunction developed, it had been over five months since he had received any cyclophosphamide. Thus, cyclophosphamide-induced toxicity is quite unlikely. Lenalidomide is associated with thromboembolism but rarely with any other cardiovascular toxicity (as compared to thalidomide which can cause bradyarrhythmias) [8, 9].

Bortezomib is a dipeptide boronate proteasome inhibitor. It reversibly binds and inhibits the 20S proteasome which is the proteolytic core particle of the 26S proteasome [10]. In the United States, it is approved for the treatment of multiple myeloma and mantle cell lymphoma. The presence of cardiac dysfunction with its use ranges from 0 to 17.9% in reported studies, though a meta-analysis of these data failed to identify definite increased cardiac risk [3]. The reported adverse cardiac events included cardiomyopathy/congestive heart failure, myocardial infarction, AV block, and supraventricular tachycardia [2, 11–13]. Some data suggests that these cardiotoxicities may occur even in the absence of underlying cardiovascular disease or risk factors. For example, one observational study reported 11.6% incidence of cardiac complications in patients without prior cardiac history treated with bortezomib [2]. The lack of prospective studies has made it difficult to accurately quantitate the true risk of cardiac dysfunction associated with bortezomib however.

The exact mechanism by which bortezomib interacts with cardiac myocardium is unknown, but there are several postulated mechanisms for the development of cardiac dysfunction, including direct cardiac insult as well as indirect exacerbation of underlying cardiac disease. The ubiquitin-proteasome pathway plays an important role in regulating the cell cycle in cardiac myocytes. As a proteasome inhibitor, bortezomib impairs the activation of transcription factors such as nuclear factor kappa-beta which is essential for cell survival, regulative apoptosis gene expression, and angiogenesis [14]. Proteasome inhibition also has downstream effects on vascular smooth muscle cells leading to atherosclerotic plaque instability [15]. In patients with cardiac risk factors or prior history of coronary artery disease, bortezomib may cause plaque progression and increase the vulnerability of existing plaque leading to ischemic events. In animal studies, there has been evidence of decreased proteasome activity impairing cardiac function by accumulation of proteins inside the myocytes as well as mitochondrial abnormalities resulting in impaired contractility [16, 17]. Through one or more of these mechanisms, the end result for patients undergoing treatment with bortezomib is increased risk of cardiac dysfunction.

Although limited data currently exist, similar cardiovascular toxicities have been associated with the use of the novel irreversible proteasome inhibitor carfilzomib. The majority of the observed cardiotoxicities with this agent were new-onset left ventricular dysfunction. These findings are most common in patients who had a history of cardiac events and exposure to doxorubicin. In this population, elevated BNP levels were also commonly observed, although the clinical significance of this finding remains unclear. In addition, hypotension and arrhythmias including atrial fibrillation have been reported [18, 19]. Given these data, in patients that develop significant cardiac dysfunction due to bortezomib or another proteasome inhibitor, considerable caution should be exercised if rechallenge with the drug is deemed necessary. If the primary cardiac toxicity has not been definitely treated (e.g., with a pacemaker) and/or completely resolved, alternative agents should be considered.

There is increasing evidence that cardiac biomarkers can both predict and detect chemotherapy-induced cardiotoxicity. Most of the research has focused on troponin and BNP. Cardiac troponins (I and T) are sensitive and specific markers of myocardial injury, and BNP is a neurohormone released from the ventricular tissue in the setting of elevated filling pressure and myocardial stress. A number of studies have evaluated the role of troponin in the detection of subclinical myocardial disease and the risk of developing overt cardiac dysfunction with the use of chemotherapeutic agents, particularly anthracyclines and trastuzumab. In a study of 211 breast cancer patients receiving anthracyclines, patients with baseline elevated troponin levels were more likely to develop cardiac dysfunction during therapy [20]. In a related study, elevations in troponin as early as 12 hours after exposure to anthracyclines predict patients at higher risk for future cardiovascular events [21]. Similarly, there was an increased risk of cardiotoxicity in breast cancer patients exposed to anthracyclines and trastuzumab who demonstrated increased troponin levels [22]. Although the majority of studies suggest that BNP is useful in predicting anthracycline induced cardiac dysfunction, the data is conflicting in the trastuzumab population [22–25]. BNP may also be useful in predicting and monitoring cardiac dysfunction due to tyrosine kinase inhibitors used in the treatment of renal cell carcinoma [26].

Biomarker elevations are also predictive of mortality and adverse outcomes in other cancer populations such as those with cardiac amyloidosis or stem cell transplant recipients. Both NT pro-BNP and troponin T can be used as markers of disease prognosis in patients with cardiac amyloidosis [27, 28]. In these patients being considered for stem cell transplant, elevated levels of troponin and BNP predict early morbidity and mortality providing guidance about a patient's suitability for this procedure [29, 30].

Despite this increasingly robust biomarker data, there are no studies specifically addressing the role of biomarkers in predicting bortezomib cardiotoxicity. BNP elevations were commonly observed in patients exposed to the proteasome inhibitor carfilzomib (median increase of 407 pg/mL from baseline); however, the predictive and/or diagnostic benefit is not clear [18]. Although the incidence of cardiotoxicity is lower with bortezomib compared to other chemotherapeutics, it still may be reasonable to apply the surveillance algorithms prior to and during therapy with proteasome inhibitors in an effort to detect those at higher risk for developing cardiac dysfunction. Using biomarkers to detect subclinical toxicity may allow for earlier cardiovascular treatment and/or intervention to prevent more significant cardiac dysfunction. Nonetheless, additional studies are necessary to specifically evaluate the potential predictive and/or diagnostic role of cardiac biomarkers in bortezomib treated patients and to identify the optimal timing and frequency of monitoring.

## 4. Conclusion

Heart block and myocardial scar/fibrosis are rare but serious side effects associated with bortezomib administration. Although the incidence of cardiotoxicity is low in patients treated with bortezomib, these events can affect patient morbidity and mortality independent of the oncologic prognosis. Improved surveillance and early detection of cardiac disorders during therapy has the potential to improve patient outcomes and prevent the discontinuation of cancer therapy.

## Figures and Tables

**Table 1:** Multiple myeloma treatment summary with relevant clinical and laboratory parameters.

	5/23/2013 Prechemotherapy	6/18/2013 VCD, 1 cycle	8/6/2013 VCD, 3 cycles Change to RVD	10/1/2013 RVD, 2 cycles	12/3/2013 RVD, 4 cycles	12/30/2013 RVD, 5 cycles
IgA (mg/dL)	1758	2358	2130	1377	885	492
M Spike (g/dL)	2.91	2.7	2.0	2.0	1.2	0.7
BUN (mg/dL)	16	22	25	19	11	11
Creatinine (mg/dL)	0.95	1.0	1.2	0.9	0.9	0.9
24-hour urine protein (g)	19.8		2.4		0.78	1.0
Blood pressure (mmHg)	127/72	119/58	108/63	120/66	107/67	117/69
Pulse (bpm)	80	60	75	63	71	69
